# Immunoglobulin M Monoclonal Gammopathies of Clinical Significance

**DOI:** 10.3389/fonc.2022.905484

**Published:** 2022-06-09

**Authors:** Louis-Pierre Girard, Cinnie Yentia Soekojo, Melissa Ooi, Wee Joo Chng, Sanjay de Mel

**Affiliations:** ^1^ Aberdeen Royal Infirmary, National Health Service Grampian, Scotland, United Kingdom; ^2^ Department of Haematology-Oncology, National University Cancer Institute, National University Health System, Singapore, Singapore; ^3^ Cancer Science Institute of Singapore, National University of Singapore, Singapore, Singapore; ^4^ Department of Medicine, Yong Loo Lin School of Medicine, National University of Singapore, Singapore, Singapore

**Keywords:** immunoglobulin M, monoclonal gammopathy of clinical significance (MGCS), monoclonal gammopathy of undetermined significance, MGRS, monoclonal gammopathy of neurological significance

## Abstract

Immunoglobulin M monoclonal gammopathy of undetermined significance (MGUS) comprises 15-20% of all cases of MGUS. IgM MGUS is distinct from other forms of MGUS in that the typical primary progression events include Waldenstrom macroglobulinaemia and light chain amyloidosis. Owing to its large pentameric structure, IgM molecules have high intrinsic viscosity and precipitate more readily than other immunoglobulin subtypes. They are also more commonly associated with autoimmune phenomena, resulting in unique clinical manifestations. Organ damage attributable to the paraprotein, not fulfilling criteria for a lymphoid or plasma cell malignancy has recently been termed monoclonal gammopathy of clinical significance (MGCS) and encompasses an important family of disorders for which diagnostic and treatment algorithms are evolving. IgM related MGCS include unique entities such as cold haemagglutinin disease, IgM related neuropathies, renal manifestations and Schnitzler’s syndrome. The diagnostic approach to, and management of these disorders differs significantly from other categories of MGCS. We describe a practical approach to the evaluation of these patients and our approach to their treatment. We will also elaborate on the key unmet needs in IgM MGCS and highlight potential areas for future research.

## Introduction

Monoclonal gammopathy of unknown significance (MGUS) is defined by a <10% plasma cell (or lymphoplasmacytic cell) bone marrow (BM) infiltrate with paraproteinaemia quantified at less than 30g/l (1). Importantly, these patients do not have symptoms or organ damage. While the majority of MGUS involve an immunoglobulin G or A clone, approximately 15-20% are associated with an immunoglobulin M (IgM) paraprotein (2). IgM MGUS is characterised by progression to Waldenstrom macroglobulinaemia (WM) or light chain amyloidosis (ALA) rather than multiple myeloma (MM) (2). Furthermore, the spectrum of clinical manifestations associated with IgM paraproteinaemias are distinct, partly owing to the large pentameric structure of IgM (3). As patients with IgM MGUS are by definition asymptomatic, watchful waiting has long been the standard of care (4).

It is recognised however, that not all patients with MGUS are asymptomatic. A subset suffer organ damage due to the paraprotein despite not fulfilling criteria for a haematologic malignancy (5). These entities were first recognised in 2005 and collectively described as IgM related disorders (IgM-RDs) by Cesana and colleagues (6). More recently, the term monoclonal gammopathy of renal significance (MGRS) was employed to describe this phenomenon in the renal context (7). Monoclonal gammopathy of clinical significance (MGCS) has since been adopted as a term which more broadly encompasses this scenario (8).

**Figure 1:**
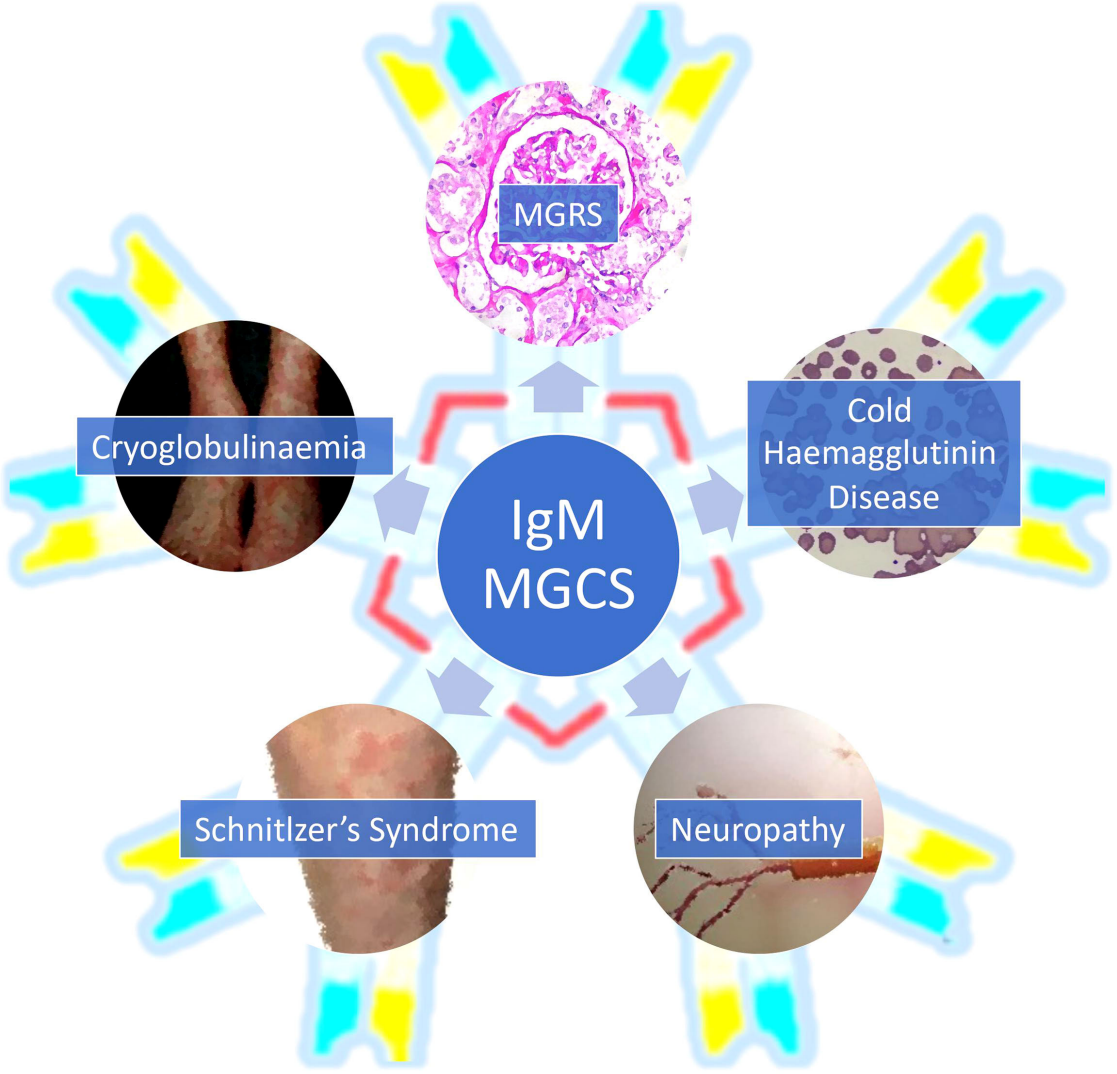
An overview of the clinical presentations associated with IgM monoclonal gammopathy of clinical significance. MGCS, monoclonal gammopathy of clinical significance; MGRS, monoclonal gammopathy of renal significance.

While any subtype of MGUS can drive an MGCS, IgM paraproteins cause unique clinical syndromes which can be challenging to diagnose and treat. IgM MGCS are notable for the high prevalence of immunologic phenomena which we discuss in detail below (3, 9). The higher prevalence of immune mediated manifestations in IgM MGCS may also be related to its multimeric structure (9). Here, we outline the key subcategories of IgM MGCS illustrated by clinical cases. We also highlight the major diagnostic and therapeutic considerations as well as the challenges and potential future developments in this field. The clinical manifestations attributable to IgM paraprotein related MGCS are summarised in **Figure 1** and an overview of IgM MGCS entities is shown in **Table 1**.

**Table 1:** An overview of the key clinical features, diagnostic and management considerations in IgM monoclonal gammopathy of clinical significance entities.

Subtype of IgM MGCS	Clinical Presentation	Diagnostic Process	Management Considerations
Neurological	Paraesthesia, painful neuropathy, ataxia. Rarely ophthalmoplegia in CANOMAD/CANDA.	Nerve conduction studies. Anti MAG, disialosyl antibodies. Neuroimaging when appropriate.	Rituximab based therapy is the first line option for anti MAG associated neuropathy. IVIG is a consideration in those who do not respond.
Cutaneous	Urticarial rash and fever in Schnitzler syndrome. Papular lesions are more common in macroglobulinosis. Ulcers and skin necrosis in cryoglobulinemia.	Skin biopsy and correlation with clinical features.	IL-1 antagonists are the first line therapy for Schnitzler syndrome. There is emerging data for IL-6 antagonists. Rituximab based therapy in selected cases.
Renal	Nephrotic syndrome. Asymptomatic rise in creatinine.	Renal biopsy and correlation with clinical features.	Rituximab based therapy for a lymphoplasmacytic clone. Bortezomib based therapy for a plasma cell driven MGCS.
IgM associated cryoglobulinaemia	Can present with skin , nerve and renal involvement. Skin lesions typically affect the extremities and non-healing ulcers can occur.	Detection of plasma cryoglobulins. Leukocytoclastic vasculitis in type 2 cryoglobulinaemia.	Rituximab based therapy should be considered for cryoglobulins with an underlying lymphoplasmacytic clone.
IgM AL Amyloidosis	Renal, neurologic and cardiac involvement are best described. Other organs can also be involved.	Histologic confirmation of amyloid. Mass spectrometry-based confirmation of the amyloid fibril subtype.	Rituximab based therapy for IgM amyloid driven by a lymphoplasmacytic clone. Bortezomib based therapy for those cases with a plasma cell clone.
IgM POEMS syndrome	Peripheral neuropathy is present in all cases. Skin lesions, endocrinopathy, organomegaly and bone lesions may also occur.	Nerve conduction studies to confirm demyelinating peripheral neuropathy. VEGF quantification Correlation with other clinical features.	No standard of care for POEMS syndrome driven by a lymphoplasmacytic clone. Rituximab based therapy to be considered. Lenalidomide based therapy for plasma cell driven cases.
Immuno-haematologic IgM MGCS	Symptomatic anaemia and acral cyanosis in primary cold agglutinin disease. Mucocutaneous bleeding in acquired VWD and ITP.	Peripheral blood film, biochemical indices of haemolysis, direct Coomb’s test and cold agglutinin titre. VWF antigen and ristocetin co factor activity. VWF multimer analysis. ITP is a diagnosis of exclusion, work up as for “idiopathic” ITP.	Rituximab based therapy for primary CAD. Rituximab monotherapy and bendamustine rituximab are considerations. No standard of care for AVWD, achievement of haemostasis and clone directed therapy to be considered. Steroid therapy as for idiopathic ITP is appropriate as first treatment. Clone directed therapy in patients who do not respond.

MGCS, monoclonal gammopathy of clinical significance; MGRS, monoclonal gammopathy of renal significance; POEMS, Polyneuropathy, Organomegaly, Endocrinopathy, Monoclonal gammopathy and Skin abnormalities; CANOMAD, Chronic Ataxic Neuropathy, Ophthalmoplegia, IgM paraprotein, Cold Agglutinins, Disialosyl antibodies; DADS-M, Distal acquired demyelinating symmetric neuropathy. MAG, Myelin associated glycoprotein. CAD, Cold agglutinin disease, VWD, Von Willebrand disease, ITP, Immune thrombocytopaenic purpura. IVIG, intravenous immunoglobulin. IL-1, Interleukin 1; IL-6, Interleukin 6. VEGF, Vascular endothelial growth factor.

## Subtypes of IgM Monoclonal Gammopathy of Clinical Significance

### Neurological IgM MGCS

#### Case Study

*A 70-year-old gentleman presented with unsteady gait and examination revealed a sensory ataxia and complex ophthalmoplegia. A magnetic resonance imaging (MRI) scan of the brain and spine showed no mass lesion and cerebrospinal fluid studies were unremarkable. A nerve conduction study showed a demyelinating peripheral neuropathy. A full blood count showed no cytopaenias although a peripheral blood smear revealed cold agglutination of the red cells. Serum immunofixation demonstrated an IgM kappa paraprotein quantified at 10g/l.*

*A whole body computed tomography (CT) scan showed no lymphadenopathy and a BM aspirate and biopsy demonstrated a lymphoplasmacytic infiltrate comprising approximately 5% of marrow nucleated cells. The MYD88 L265P mutation was not detected by allele specific PCR. There was no evidence of anti-myelin associated glycoprotein (MAG) antibodies, while anti ganglioside antibodies (GD1b subtype) were detected. A diagnosis of CANOMAD (chronic ataxic neuropathy, ophthalmoplegia, IgM paraprotein, cold agglutinins, and disialosyl antibodies) syndrome was made based on these findings. The patient was treated with rituximab 375mg/m2 weekly for four weeks and made a partial neurological recovery despite normalisation of the serum IgM level and immunofixation becoming negative.*

Paraproteinaemic neuropathy in the absence of an active B-cell or plasma cell malignancy is well described and has been established as an MGCS entity (8, 10). The monoclonal protein binding to neural antigens including MAG, ganglioside, and ganglio-N-tetraosylceramide proteins have been proposed as the mechanism behind neurologic MGCS (5, 11). The finding of monoclonal protein deposits within myelin fibres and Schwann cells has supported this hypothesis (5). Interestingly, IgM paraproteins account for a disproportionate 60% of monoclonal gammopathy related neuropathies (12).

Anti MAG neuropathy is the best characterized among IgM associated neuropathies and is more commonly associated with IgM MGUS than WM (13). The titre of anti MAG antibodies has also been shown to impact the clinical presentation and response to treatment (13). Distal acquired demyelinating symmetrical neuropathy with a monoclonal protein (DADS-M) is also associated with IgM gammopathies and manifests as a progressive and symmetrical distal neuropathy, which can affect both sensory and motor functions (5, 12). Findings on nerve conduction studies resemble demyelination, and nerve biopsies may reveal axonal loss (10, 12).

As illustrated by the case study above, neurological manifestations of IgM paraproteins can go beyond peripheral neuropathy. The CANOMAD syndrome is a rare example of neurological IgM MGCS characterized not only by chronic sensory ataxia but also ophthalmoplegia and cold agglutinins (14). Not all the manifestations of CANOMAD are seen in every patient, hence an alternative terminology CANDA (chronic ataxic neuropathy with disialosyl antibodies) has been proposed, highlighting the key features of chronic ataxia and IgM antibodies targeting gangliosides rather than MAG (14). CANOMAD/CANDA is characterised by IgM antibodies against disialosyl epitopes on gangliosides GD1b, GT1c, GQ1b and GD3I (15). These typically affect large sensory fibres resulting in the clinical manifestations described above (15). Nerve conduction studies and histopathology can show demyelinating and axonal features (15).

IgM paraproteins can also lead to peripheral neuropathy *via* type I cryoglobulinaemia, manifesting in approximately 20-40% of patients (16). Cryoglobulinaemic neuropathy is caused by cryoglobulin deposition leading to a small vessel vasculitis, often occurring at cold temperatures and affecting the extremities (17). The typical presentation is that of a painful sensory neuropathy although motor involvement has also been reported (16, 17). IgM related cryoglobulins are discussed further under “immunohaematologic IgM MGCS”.

Given the complexity and variety of IgM associated neuropathies, a detailed history, complete neurological examination, nerve conduction studies and serologic evaluation for antibodies against MAG or gangliosides are key to reaching an accurate diagnosis. It is particularly important not to miss IgM associated multi system syndromes causing neuropathy. These include IgM ALA, polyneuropathy, organomegaly, endocrinopathy, monoclonal gammopathy and skin changes (POEMS) syndrome and cryoglobulin related neuropathies. The treatment for these conditions differs significantly from that for Anti MAG neuropathy, DADS-M and CANOMAD/CANDA (12). It is also vital to exclude peripheral neuropathies related to other medical causes which may be easily reversed by standard therapies. Making this distinction can however be challenging based on both clinical assessment and nerve conduction studies (12).

The optimal management for neurological IgM MGCS remains controversial due to its rarity and lack of prospective clinical trials in this field (18). Intravenous immunoglobulin (IVIG) might be considered, though it is possible responses maybe short lived (14, 19). Le Cann et al. proposed IVIG as the first line treatment option for CANOMAD/CANDA based on responses seen in a large retrospective series (14). IVIG has also been suggested as a treatment for DADS-M while more data on the frequency of clinical responses are awaited (5).

Rituximab is one of the best studied therapies for IgM neuropathies and reduces the circulating paraprotein by targeting the B-cell clone (11, 20, 21). Importantly, the reduction in IgM was shown to correlate with neurological improvement in some patients (14). Rituximab has well documented efficacy in Anti MAG neuropathies based on retrospective studies (11). Although transient worsening of the neuropathy was noted in a minority of patients, clinical improvements occurred in over 30% and were more common in patients who had a reduction in Anti MAG titres (13, 22).

Although plasmapheresis is a recognised treatment modality for hyper viscosity associated with WM (23), its role in IgM MGCS remains less certain. The use of Plasmapheresis was reported in a small number of patients with CANOMAD/CANDA; however, it is yet to be established as a first line treatment for any subtype of neurologic MGCS (5, 12, 24). A single case-series has suggested fludarabine as an option, however given the toxicity of this drug its use may not be justified in the context of a low level paraprotein (25). Deeper insights into the biology of neurological IgM MGCS are necessary in order to better define the optimal diagnostic strategy and treatment for these patients.

### Cutaneous IgM MGCS

#### Case Study

*A 60-year-old gentleman was referred to the dermatology clinic with persistent urticarial rashes on the trunk and extremities. The rashes had been occurring intermittently for over 3 years and had more recently been accompanied by fevers. Physical examination revealed an urticarial rash but no lymphadenopathy or hepatosplenomegaly. His full blood count was normal while serum immunofixation detected a monoclonal IgM kappa paraprotein quantified at 5g/l. BM studies showed a lymphoplasmacytic infiltrate comprising 3% of nucleated cells while a computed tomography (CT) scan of the whole body showed no organomegaly.*

*A skin biopsy showed a neutrophilic urticarial dermatosis characteristic of Schnitzler syndrome. As his symptoms were mild, a watch and wait strategy was initially employed. However, his skin symptoms progressed over the next year although his IgM level remained stable. He was treated with a course of anti-interleukin 1 therapy (canakinumab) with a good clinical response.*

A variety of cutaneous eruptions may be associated with paraproteinaemias, ranging from relatively benign conditions such as xanthoderma, to more destructive lesions like pyoderma gangrenosum (26, 27). Paraprotein related skin lesions occurring in the absence of an active B-cell or plasma cell neoplasm are termed monoclonal gammopathy of cutaneous significance (26). While intact proteins may be deposited in the skin as entire immunoglobulin molecules (as in macroglobulinosis), they can also occur as cutaneous light-chain or heavy-chain deposition diseases (28). Deposition of modified proteins may be in the form of β-pleated sheets (in ALA), cryoprecipitated immunoglobulin (in cryoglobulinaemic vasculopathy), or crystallised deposits (in crystal storing histiocytosis) (28).

Macroglobulinosis and Schnitzler syndrome are the dermatoses specifically associated with IgM paraproteins (3, 29, 30). Macroglobulinosis is characterised by skin-coloured papules on the extensor surfaces of the limbs and cutaneous IgM deposition is a hallmark of this condition (30). Urticarial rash with a neutrophilic urticarial dermatosis seen on histopathology are the key dermatologic features of Schnitzler syndrome (29). Hepatosplenomegaly, bone pain due to osteosclerotic lesions and lymphadenopathy may occur as extra cutaneous manifestations (29). A deregulated cutaneous inflammasome has been proposed to play a key part in the pathogenesis of Schnitzler syndrome. While interleukin 6 (IL-6) and IL-8 were increased in these patients (31, 32), further studies are needed to unravel the cross talk between the IgM paraprotein and the deregulated cytokine milieu (33).

Cutaneous manifestations are also a common feature of cryoglobulinaemia and may occur due to a small vessel vasculitis and vascular occlusion by the cryoglobulin (16). These are more common in the extremities and include purpura, livedo reticularis, Raynaud phenomenon and ulcers (16, 34). Skin necrosis and ulcers can cause significant morbidity as they are slow to heal and can even result in gangrene (34). IgM related cryoglobulinaemia is discussed further under the section “immunohaematologic IgM MGCS”.

As with other subtypes of MGCS, the optimal treatment for cutaneous MGCS remains uncertain. A conservative approach is appropriate if the skin manifestations are not causing significant symptoms (8). With regard to clone directed therapy, rituximab would be a consideration for a lymphoplasmacytic clone and bortezomib an option for plasma cell driven MGCS (5, 8). Targeting the pro-inflammatory milieu through IL-1 antagonists (as has been demonstrated in Schnitzler syndrome), may be an alternative to clone directed therapy (31). Although anti IL-1 therapy is considered the therapy of choice for Schnitzler syndrome, there is emerging evidence that a subset of patients may respond to IL-6 antagonists instead (35). These data suggest that the cytokine dysregulation in Schnitzler syndrome is heterogeneous, and future studies should focus on evaluating biomarkers for response to interleukin antagonists.

These data underscore the need for a better appreciation of the pathophysiology of IgM associated skin lesions. This will be essential for the development of more effective targeted therapeutics these disorders.

### IgM Monoclonal Gammopathies of Renal Significance

#### Case Study

*A 50-year-old lady presented with nephrotic range proteinuria and oedema. Her serum creatinine was raised at 150 μmol/l while her full blood count and calcium were normal. Serum immunofixation demonstrated an IgM kappa paraprotein quantified at 4g/l. A BM aspirate and trephine biopsy showed 5% clonal lymphoplasmacytic cells and a CT scan showed no lymphadenopathy or organomegaly. A renal biopsy showed proliferative glomerulonephritis with monoclonal IgM deposits. She was treated with rituximab monotherapy resulting in normalisation of her serum IgM (although immunofixation remained weakly positive). Her renal function improved and stabilised over the next 6 months.*

In patients with a paraprotein and renal impairment, the challenge is to determine whether the renal injury can be attributed to the paraprotein or to other medical conditions. As renal impairment is a defining feature of MM and can occur in other monoclonal gammopathies, it is imperative to first exclude a plasma cell or B-cell lymphoproliferative disorder (LPD) (7, 36). MGRS are subclassified into organised and non-organised categories based on the histopathologic patterns of renal immunoglobulin deposition (7). A renal biopsy is hence recommended to diagnose and subclassify MGRS, as well as to exclude other causes of renal impairment (7, 37). Type II cryoglobulinaemic vasculitis and proliferative glomerulonephritis with monoclonal immunoglobulin deposits are the subtypes of MGRS described to have renal IgM deposits (7). ALA, type 1 cryoglobulinaemic glomerulonephritis, crystal storing histiocytosis and immunotactoid glomerulonephritis can also arise in the context of an IgM paraprotein (7, 37).

The diagnosis of MGRS requires significant expertise and electron microscopy is recommended in the diagnostic process (7). As diagnosing MGRS can be challenging outside of specialised centres, referral to tertiary centres with the infrastructure to make this diagnosis is prudent. The need for a renal biopsy to diagnose MGRS can also be a challenge as these patients are often elderly and have co-morbidities which increase procedural risk. Meticulous clinical assessment, along with close follow up and communication is required in these challenging situations.

Although it is accepted that the treatment for MGRS should be clone directed, there is a paucity of prospective trials to inform the optimal choice of therapy (5, 37). While MGRS treatment regimens are usually extrapolated from MM and other indolent B-cell LPD, the choice of treatment for MGRS occurring with an IgM paraprotein would usually necessitate targeting a lymphoplasmacytic rather than a plasma cell clone (37). We also propose that the treatment for MGRS need not be as intensive as for an active MM or B-LPD given that we are by definition, dealing with a smaller clonal burden. It is imperative that future research focuses on the biology of MGRS as well as clinical trials with a view to identifying less toxic but effective treatment modalities.

### Immuno-Haematologic IgM MGCS

#### Case Study

*A 75-year-old gentleman was referred for haematology consultation when he presented with symptomatic anaemia and acral cyanosis. Physical examination was unremarkable. His full blood count showed a haemoglobin of 8g/dl with peripheral blood findings of agglutination resolving on warming to 37° C. The direct Coomb’s test was positive for the complement factor C3b while being negative for IgG. Lactate dehydrogenase, reticulocyte count and unconjugated bilirubin were elevated. His cold agglutinin titre was 70 and the thermal amplitude was 29° C. Serum immunofixation revealed an IgM kappa paraprotein quantified at 2g/l. BM studies showed increased erythropoiesis with a lymphoplasmacytic infiltrate comprising 5% of nucleated cells. He was treated with single agent rituximab 375mg/m2 weekly for 4 weeks and achieved a normalisation of his haemoglobin as well as haemolytic indices.*

Primary cold agglutinin disease (CAD) is characterized by an IgM antibody binding to the red blood cell membrane and is associated with a clonal lymphoplasmacytic infiltrate in the bone marrow (38–41). The histopathological features and absence of MYD88 mutations suggests that the B-cell infiltration associated with CAD is distinct from that of WM (38). The antibody found in patients with CAD typically binds to the red cell antigen I, resulting in agglutination and in some cases haemolysis *via* complement activation (38, 42). Patients with cold agglutinins and no active haemolysis can be monitored without treatment and cold avoidance should be advised (39, 42, 43).

For those with active haemolysis, rituximab has traditionally been the first line treatment and results in response rates of close to 50% as monotherapy (42) (44). The combination of bendamustine rituximab can yield response rates of over 70% albeit with higher rates of haematologic toxicity compared to rituximab alone (43, 45). More recently, inhibition of the complement pathway through sutimlimab has shown promising efficacy in CAD (46). Larger studies evaluating this agent are eagerly awaited.

Cold temperatures can also result in cryoglobulinaemia among some patients with IgM paraproteins (16). Cryoglobulins precipitate at low temperatures, causing endothelial damage and small- to medium vessel vasculitis (47). Raynaud phenomenon, acrocyanosis, urticaria, peripheral neuropathy or renal failure can all be a result of cryoglobulin precipitation (16). Type I cryoglobulinaemia is typically associated with monoclonal gammopathies of the IgM or IgG subtypes which are seen in about 40% of patients. Type 2 cryoglobulinaemia is characterised by a combination of monoclonal IgM with rheumatoid factor activity and polyclonal IgG (16). A leukocytoclastic vasculitis is a typical histopathological finding in type 2 cryoglobulinaemia. Both subtypes can cause immune-complex mediated vasculitis and skin ulcers (16). The management of cryoglobulinaemias is also based on limited evidence with clone directed therapy to be considered in those driven by a paraprotein (16, 47).

Acquired Von Willebrand disease (AVWD) has been described as a rare immune-haematologic manifestation of IgM gammopathies (48). Among the mechanisms proposed for AVWD in monoclonal gammopathies include von Willebrand factor (VWF) specific antibodies (49) and the VWF multimers being absorbed onto the lymphoplasmacytic cells (50). Accelerated degradation of VWF has also been proposed as a potential cause of AVWD in addition to immune complex formation between VWF and non-specific antibodies (51, 52). The clinical presentation is similar to that of other subtypes of VWD and the evidence base for management is limited (51). Treatment is based on achieving haemostasis as well as targeting the underlying B-cell clone (51, 52).

Immune thrombocytopaenic purpura (ITP) is another rare immunohaematologic phenomenon reported with IgM paraproteins (9, 53). The mechanism is thought to be an immune mediated platelet destruction triggered by the IgM secreting clone (53). While the clinical presentation is similar to that of “idiopathic” ITP, the optimal management remains an area of uncertainty (53, 54). Rare responses to clone directed therapy have been reported and are worthy of further evaluation in patients who do not respond to steroids (54).

### Multi System Disorders Associated With IgM Paraproteins

#### Case Study

*A 50-year-old gentleman was admitted for heart failure with a normal coronary angiogram. Echocardiographic and cardiac MRI features were suspicious for cardiac amyloidosis. Serum immunofixation showed an IgM kappa paraprotein quantified at 6 g/l and BM studies confirmed a lymphoplasmacytic infiltrate comprising 7% of nucleated cells. The MYD88 L265P was detected using allele specific PCR of CD19 selected BM cells and an abdominal fat pad aspiration provided histologic confirmation of light chain amyloidosis. Mass spectrometry was used to confirm that the amyloid was of the light chain sub-type. A CT scan showed no lymphadenopathy, and his full blood count and renal function were normal. The patient was treated with six cycles of bendamustine-rituximab and achieved a complete haematologic response, unfortunately a cardiac response has not been achieved as of present.*

While the previous sections focused on organ specific disorders driven by small IgM secreting clones, it is essential that multi system syndromes are not overlooked in this context. ALA is a potentially life-threatening condition which is more commonly associated with non IgM paraproteins (55). ALA associated with IgM paraproteins has been proposed as a distinct entity with a higher prevalence of soft tissue and nerve involvement compared to other subtypes (56). Although cardiac involvement is reported to be less common in IgM ALA, an electrocardiogram, serum troponin, NT-pro BNP and an echocardiogram remain essential to exclude cardiac disease which has major implications for management (57).

While biopsy of the involved organ is a consideration, this carries risk and bleeding complications are reported more frequently in patients with renal amyloidosis undergoing biopsy (58). Abdominal fat pad aspirates may therefore be a safer initial option with high sensitivity and specificity for the histologic diagnosis of ALA (55). As approximately 25-30% of amyloid cannot be accurately subtyped by immunohistochemistry, mass spectrometry based amyloid subtyping may be required and remains the gold standard (55).

IgM paraproteins are also rarely described to drive POEMS syndrome (59). Interestingly, both ALA and POEMS are more commonly associated with IgM lambda than IgM Kappa paraproteins (59). Neuropathy is a major diagnostic criterion for POEMS syndrome and is typically an ascending sensorimotor demyelinating peripheral neuropathy (5, 60). The neuropathy is often painful while hyperaesthesia is also reported (61). The pathophysiology of POEMS related neuropathy is incompletely understood and possibly involves alterations of cation transport in the nodes of Ranvier as well vascular endothelial growth factor mediated vasculopathy (60).

The management of IgM ALA and POEMS are areas of great uncertainty, as the bulk of evidence for these diseases come from trials of patients with non-IgM ALA driven by plasma cell clones. Given the rarity of these disorders, we currently rely on case reports and case series describing rituximab based chemo-immunotherapy which results in haematologic responses but not always an organ response (56).

### General Considerations in the Diagnosis and Evaluation of IgM MGCS

Distinguishing IgM MGCS from WM is important and can be achieved based on the presence of symptoms attributable to the IgM clone and establishing the diagnosis of WM as defined by the world health organisation 2016 classification (62). While patients with smouldering WM are also asymptomatic, they have a greater than 10% lymphoplasmacytic infiltrate and/or a paraprotein quantification of greater than 30g/l (63). IgM MM is a rare but important differential diagnosis to exclude (64). This distinction is usually possible based on MM specific clinicopathological features and the finding of immunoglobulin heavy chain translocations which are not described in WM or IgM MGUS (64).

IgM MGCS entities are rare and many of these disorders have non-specific symptoms which could be attributed to concomitant medical disorders. Having a high index of suspicion and considering the diagnosis of IgM MGCS is therefore a crucial first step in the evaluation process. The importance of interdisciplinary collaboration cannot be emphasized enough in the diagnosis of MGCS. As illustrated by the cases above, haematologists would need to collaborate with neurologists, dermatologists, nephrologists and pathologists among others in the process of making a diagnosis. Especially for the complex multisystem disorders, a multidisciplinary approach is also required when it comes to management decision making.

**Figure 2:**
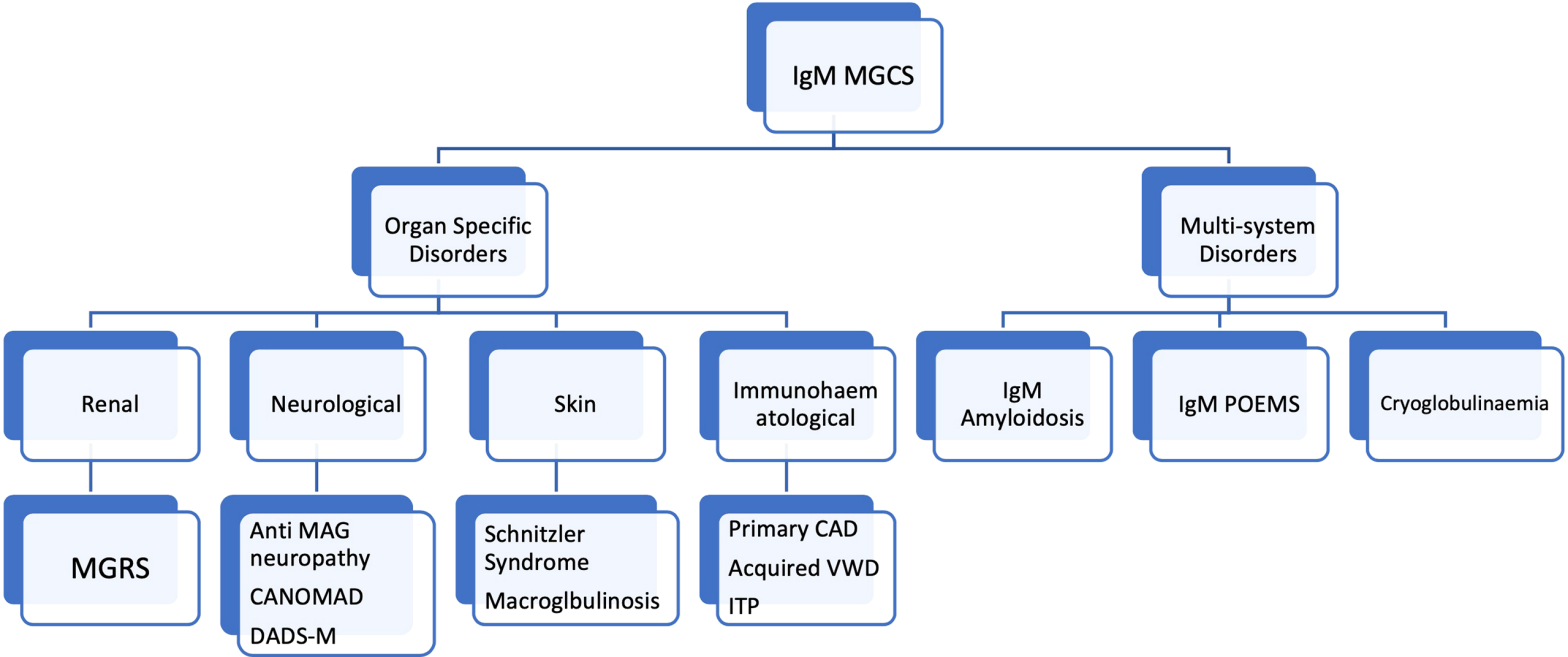
Classification of of IgM monoclonal gammopathy of clinical significance entities. MGCS, monoclonal gammopathy of clinical significance; MGRS, monoclonal gammopathy of renal significance; POEMS, Polyneuropathy, Organomegaly, Endocrinopathy, Monoclonal gammopathy and Skin abnormalities; CANOMAD, Chronic Ataxic Neuropathy, Ophthalmoplegia, IgM paraprotein, Cold Agglutinins, Disialosyl antibodies; DADS-M, Distal acquired demyelinating symmetric neuropathy. MAG, Myelin associated glycoprotein. CAD, Cold agglutinin disease, VWD, Von Willebrand disease, ITP, Immune thrombocytopaenic purpura.

Careful integration of clinical and laboratory (clinical chemistry, pathology and haematology) features is also a vital aspect of the diagnostic process. *MYD88 L265P* mutations are less frequent in IgM MGUS (approximately 60% of cases) than they are in WM (65, 66). The frequency of this mutation in IgM MGCS remains unknown but is likely to be less common than in WM given that IgM MGCS is usually associated with a lower-level B-cell infiltrate more akin to IgM MGUS. MYD88 mutations may even be absent in some scenarios such as primary CAD (38, 43). The absence of MYD88 mutations in the context of IgM MGCS should therefore not be surprising and should not put the clinician off making the diagnosis of a IgM producing B-cell clone. A suggested classification of IgM MGCS entities is outlined in **Figure 2**.

### Conclusions and Future Directions

We are only beginning to learn about the clinical manifestations of small IgM secreting clones and the resulting MGCS syndromes. We propose that multidisciplinary diagnostic and therapeutic approaches will be essential in tackling these diseases while clinical and translational research must focus on unravelling their biology and therapeutic susceptibilities. The treatment for IgM MGCS should be stratified based on the organ (s) involved, symptoms and patient fitness. While rituximab-based therapies have been established for certain subtypes of MGCS, the management for many remain uncertain. Bruton tyrosine kinase (BTK) inhibition is currently a standard treatment modality for WM, however there is limited data supporting BTK inhibitors in IgM MGCS (67, 68). The fact that the majority of IgM MGCS are likely to be MYD88 wild type suggests that the efficacy of BTK inhibition may not be as promising in IgM MGCS as it has been in WM (69, 70). A deeper understanding of the mutational profile of the lymphoplasmacytic clone in MGCS (which maybe distinct from WM) could prove clinically valuable.

Hexameric IgM has been proposed to be more immunogenic than the pentameric form, however its role in IgM gammopathies remains uncertain (71, 72). The prevalence of hexameric IgM in the context of IgM MGCS and its clinical implications should be the subject of future research. Future studies should also aim to interrogate the immune microenvironment of the organs involved as well as the BM of patients with IgM MGCS. It is possible that immune dysregulation may explain why only a subset of MGUS patients develop IgM MGCS, a finding that may have therapeutic implications.

## Author Contributions

SdM and L-PG conceptualised the study. L-PG and SdM wrote the first draft of the manuscript. WJC, MO, and CYS provided critical editing and input for the manuscript. All authors contributed to the article and approved the submitted version.

## Conflict of Interest

The authors declare that the research was conducted in the absence of any commercial or financial relationships that could be construed as a potential conflict of interest.

## Publisher’s Note

All claims expressed in this article are solely those of the authors and do not necessarily represent those of their affiliated organizations, or those of the publisher, the editors and the reviewers. Any product that may be evaluated in this article, or claim that may be made by its manufacturer, is not guaranteed or endorsed by the publisher.

## References

[1] Bird J Behrens J Westin J Turesson I Drayson M Beetham R. UK Myeloma Forum (UKMF) and Nordic Myeloma Study Group (NMSG): Guidelines for the Investigation of Newly Detected M-Proteins and the Management of Monoclonal Gammopathy of Undetermined Significance (MGUS). Br J Haematol (2009) 147:22–42. doi: 10.1111/j.1365-2141.2009.07807.x 19673884

[2] Kyle RA Benson J Larson D Therneau T Dispenzieri A Melton Iii LJ. IgM Monoclonal Gammopathy of Undetermined Significance and Smoldering Waldenström's Macroglobulinemia. Clin Lymphoma Myeloma (2009) 9:17–8. doi: 10.3816/CLM.2009.n.002 PMC3773469 19362962

[3] Girard L-P Soekojo CY Ooi M Poon LM Chng W-J de Mel S. Immunoglobulin M Paraproteinaemias. Cancers (2020) 12:1688. doi: 10.3390/cancers12061688 PMC7352433 32630470

[4] Kyle RA Durie BG Rajkumar SV Landgren O Blade J Merlini G. Monoclonal Gammopathy of Undetermined Significance (MGUS) and Smoldering (Asymptomatic) Multiple Myeloma: IMWG Consensus Perspectives Risk Factors for Progression and Guidelines for Monitoring and Management. Leukemia (2010) 24:1121–7. doi: 10.1038/leu.2010.60 PMC7020664 20410922

[5] Dispenzieri A. Monoclonal Gammopathies of Clinical Significance. Hematology (2020) 2020:380–8. doi: 10.1182/hematology.2020000122 PMC7727544 33275738

[6] Cesana C Barbarano L Miqueleiz S Lucchesini C Ricci F Varettoni M. Clinical Characteristics and Outcome of Immunoglobulin M-Related Disorders. Clin Lymphoma (2005) 5:261–4. doi: 10.3816/CLM.2005.n.012 15794861

[7] Leung N Bridoux F Batuman V Chaidos A Cockwell P D'Agati VD. The Evaluation of Monoclonal Gammopathy of Renal Significance: A Consensus Report of the International Kidney and Monoclonal Gammopathy Research Group. Nat Rev Nephrol (2019) 15:45–59. doi: 10.1038/s41581-018-0077-4 30510265 PMC7136169

[8] Fermand JP Bridoux F Dispenzieri A Jaccard A Kyle RA Leung N. Monoclonal Gammopathy of Clinical Significance: A Novel Concept With Therapeutic Implications. Blood (2018) 132:1478–85. doi: 10.1182/blood-2018-04-839480 30012636

[9] Paludo J Ansell S. Advances in the Understanding of IgM Monoclonal Gammopathy of Undetermined Significance. F1000Research (2017) 6:2142. doi: 10.12688/f1000research.12880.1 29399323 PMC5785715

[10] Rison RA Beydoun SR. Paraproteinemic Neuropathy: A Practical Review. BMC Neurol (2016) 16:13. doi: 10.1186/s12883-016-0532-4 26821540 PMC4731930

[11] Steck AJ. Anti-MAG Neuropathy: From Biology to Clinical Management. J Neuroimmunol (2021) 361:577725. doi: 10.1016/j.jneuroim.2021.577725 34610502

[12] Chaudhry HM Mauermann ML Rajkumar SV. Monoclonal Gammopathy-Associated Peripheral Neuropathy: Diagnosis and Management. Mayo Clin Proc (2017) 92:838–50. doi: 10.1016/j.mayocp.2017.02.003 PMC5573223 28473042

[13] Svahn J Petiot P Antoine JC Vial C Delmont E Viala K. Anti-MAG Antibodies in 202 Patients: Clinicopathological and Therapeutic Features. J Neurol Neurosurg Psychiatry (2018) 89:499–505. doi: 10.1136/jnnp-2017-316715 29070644

[14] Le Cann M Bouhour F Viala K Simon L Tard C Rossi C. CANOMAD: A Neurological Monoclonal Gammopathy of Clinical Significance That Benefits From B-Cell–Targeted Therapies. Blood (2020) 136:2428–36. doi: 10.1182/blood.2020007092 32959046

[15] Willison HJ O'Leary CP Veitch J Blumhardt LD Busby M Donaghy M. The Clinical and Laboratory Features of Chronic Sensory Ataxic Neuropathy With Anti-Disialosyl IgM Antibodies. Brain (2001) 124:1968–77. doi: 10.1093/brain/124.10.1968 11571215

[16] Muchtar E Magen H Gertz MA. How I Treat Cryoglobulinemia. Blood (2017) 129:289–98. doi: 10.1182/blood-2016-09-719773 27799164

[17] Gemignani F Brindani F Alfieri S Giuberti T Allegri I Ferrari C. Clinical Spectrum of Cryoglobulinaemic Neuropathy. J Neurology Neurosurg Psychiatry (2005) 76:1410–4. doi: 10.1136/jnnp.2004.057620 PMC1739369 16170087

[18] D'Sa S Kersten MJ Castillo JJ Dimopoulos M Kastritis E Laane E. Investigation and Management of IgM and Waldenström-Associated Peripheral Neuropathies: Recommendations From the IWWM-8 Consensus Panel. Br J Haematol (2017) 176:728–42. doi: 10.1111/bjh.14492 28198999

[19] Leger JM Younes-Chennoufi AB Chassande B Davila G Bouche P Baumann N. Human Immunoglobulin Treatment of Multifocal Motor Neuropathy and Polyneuropathy Associated With Monoclonal Gammopathy. J neurol neurosurg Psychiatry (1994) 57 Suppl:46–9. doi: 10.1136/jnnp.57.Suppl.46 PMC1016725 7964853

[20] Zivković SA. Rituximab in the Treatment of Peripheral Neuropathy Associated With Monoclonal Gammopathy. Expert Rev Neurother (2006) 6:1267–74. doi: 10.1586/14737175.6.9.1267 17009914

[21] Goldfarb AR Weimer LH Brannagan TH3. Rituximab Treatment of an IgM Monoclonal Autonomic and Sensory Neuropathy. Muscle Nerve (2005) 31:510–5. doi: 10.1002/mus.20244 15685616

[22] Hänggi P Aliu B Martin K Herrendorff R Steck AJ. Decrease in Serum Anti-MAG Autoantibodies Is Associated With Therapy Response in Patients With Anti-MAG Neuropathy: Retrospective Study. Neurol Neuroimmunol Neuroinflamm (2022) 9:1109. doi: 10.1212/NXI.0000000000001109 PMC8587733 34759022

[23] Gertz MA. Acute Hyperviscosity: Syndromes and Management. Blood (2018) 132:1379–85. doi: 10.1182/blood-2018-06-846816 PMC6161773 30104220

[24] Dyck PJ Low PA Windebank AJ Jaradeh SS Gosselin S Bourque P. Plasma Exchange in Polyneuropathy Associated With Monoclonal Gammopathy of Undetermined Significance. New Engl J Med (1991) 325:1482–6. doi: 10.1056/NEJM199111213252105 1658648

[25] Wilson HC Lunn MP Schey S Hughes RA. Successful Treatment of IgM Paraproteinaemic Neuropathy With Fludarabine. J Neurol Neurosurg Psychiatry (1999) 66:575–80. doi: 10.1136/jnnp.66.5.575 PMC1736362 10209166

[26] Lipsker D. Monoclonal Gammopathy of Cutaneous Significance: Review of a Relevant Concept. J Eur Acad Dermatol Venereol (2017) 31:45–52. doi: 10.1111/jdv.13847 27501129

[27] George C Deroide F Rustin M. Pyoderma Gangrenosum - A Guide to Diagnosis and Management Clinical Medicine. Lond Engl (2019) 19:224–8. doi: 10.7861/clinmedicine.19-3-224 PMC6542232 31092515

[28] Alegría-Landa V Cerroni L Kutzner H Requena L. Paraprotein Deposits in the Skin. J Am Acad Dermatol (2017) 77:1145–58. doi: 10.1016/j.jaad.2017.07.039 28985955

[29] Lipsker D. The Schnitzler Syndrome. Orphanet J Rare Dis (2010) 5:1145–1158. doi: 10.1186/1750-1172-5-38 PMC3018454 21143856

[30] Camp BJ Magro CM. Cutaneous Macroglobulinosis: A Case Series. J Cutan Pathol (2012) 39:962–70. doi: 10.1111/j.1600-0560.2012.01983.x 22882527

[31] Rowczenio DM Pathak S Arostegui JI Mensa-Vilaro A Omoyinmi E Brogan P. Molecular Genetic Investigation, Clinical Features, and Response to Treatment in 21 Patients With Schnitzler Syndrome. Blood (2018) 131:974–81. doi: 10.1182/blood-2017-10-810366 PMC5877784 29284595

[32] Masson Regnault M Frouin E Jéru I Delwail A Charreau S Barbarot S. Cytokine Signature in Schnitzler Syndrome: Proinflammatory Cytokine Production Associated to Th Suppression. Front Immunol (2020) 11. doi: 10.3389/fimmu.2020.588322 PMC7726442 33324407

[33] van Leersum FS Potjewijd J van Geel M Steijlen PM Vreeburg M. Schnitzler’s Syndrome - A Novel Hypothesis of a Shared Pathophysiologic Mechanism With Waldenström’s Disease. Orphanet J Rare Dis (2019) 14:151. doi: 10.1186/s13023-019-1117-2 31228950 PMC6589170

[34] Giuggioli D Manfredi A Lumetti F Sebastiani M Ferri C. Cryoglobulinemic Vasculitis and Skin Ulcers. Our Therapeutic Strategy and Review of the Literature. Semin Arthritis Rheum (2015) 44:518–26. doi: 10.1016/j.semarthrit.2014.10.004 25547031

[35] Krause K Feist E Fiene M Kallinich T Maurer M. Complete Remission in 3 of 3 Anti-IL-6-Treated Patients With Schnitzler Syndrome. J Allergy Clin Immunol (2012) 129:848–50. doi: 10.1016/j.jaci.2011.10.031 22154381

[36] Rajkumar SV Dimopoulos MA Palumbo A Blade J Merlini G Mateos MV. International Myeloma Working Group Updated Criteria for the Diagnosis of Multiple Myeloma. Lancet Oncol (2014) 15:e538–48. doi: 10.1016/S1470-2045(14)70442-5 25439696

[37] Jain A Haynes R Kothari J Khera A Soares M Ramasamy K. Pathophysiology and Management of Monoclonal Gammopathy of Renal Significance. Blood Adv (2019) 3:2409–23. doi: 10.1182/bloodadvances.2019031914 PMC6693003 31409583

[38] Ulla R Gunhild T Anne T Chloé S Abdirashid W Klaus B. Primary Cold Agglutinin-Associated Lymphoproliferative Disease: A B-Cell Lymphoma of the Bone Marrow Distinct From Lymphoplasmacytic Lymphoma. Haematologica (2014) 99:497–504. doi: 10.3324/haematol.2013.091702 24143001 PMC3943313

[39] Jäger U Barcellini W Broome CM Gertz MA Hill A Hill QA. Diagnosis and Treatment of Autoimmune Hemolytic Anemia in Adults: Recommendations From the First International Consensus Meeting. Blood Rev (2020) 41:100648. doi: 10.1016/j.blre.2019.100648 31839434

[40] Berentsen S Barcellini W. Autoimmune Hemolytic Anemias. N Engl J Med (2021) 385:1407–19. doi: 10.1056/NEJMra2033982 34614331

[41] Hill QA Hill A Berentsen S. Defining Autoimmune Hemolytic Anemia: A Systematic Review of the Terminology Used for Diagnosis and Treatment. Blood Adv (2019) 3:1897–906. doi: 10.1182/bloodadvances.2019000036 PMC6595261 31235526

[42] Swiecicki PL Hegerova LT Gertz MA. Cold Agglutinin Disease. Blood (2013) 122:1114–21. doi: 10.1182/blood-2013-02-474437 23757733

[43] Berentsen S. How I Treat Cold Agglutinin Disease. Blood (2021) 137:1295–303. doi: 10.1182/blood.2019003809 33512410

[44] Berentsen S Tjønnfjord GE Brudevold R Gjertsen BT Langholm R Løkkevik E. Favourable Response to Therapy With the Anti-CD20 Monoclonal Antibody Rituximab in Primary Chronic Cold Agglutinin Disease. Br J Haematol (2001) 115:79–83. doi: 10.1046/j.1365-2141.2001.03078.x 11722415

[45] Berentsen S Randen U Oksman M Birgens H Tvedt THA Dalgaard J. Bendamustine Plus Rituximab for Chronic Cold Agglutinin Disease: Results of a Nordic Prospective Multicenter Trial. Blood (2017) 130:537–41. doi: 10.1182/blood-2017-04-778175 28533306

[46] Röth A Barcellini W D’Sa S Miyakawa Y Broome CM Michel M. Sutimlimab in Cold Agglutinin Disease. New Engl J Med (2021) 384:1323–34. doi: 10.1056/NEJMoa2027760 33826820

[47] Roccatello D Saadoun D Ramos-Casals M Tzioufas AG Fervenza FC Cacoub P. Cryoglobulinaemia. Nat Rev Dis Primers (2018) 4:11. doi: 10.1038/s41572-018-0009-4 30072738

[48] Mayerhofer M Haushofer A Kyrle PA Chott A Müllner C Quehenberger P. Mechanisms Underlying Acquired Von Willebrand Syndrome Associated With an IgM Paraprotein. Eur J Clin Invest (2009) 39:833–6. doi: 10.1111/j.1365-2362.2009.02177.x 19572993

[49] Coucke L Marcelis L Deeren D Van Dorpe J Lambein K Devreese K. Lymphoplasmacytic Lymphoma Exposed by Haemoptysis and Acquired Von Willebrand Syndrome. Blood Coagulation Fibrinolysis (2014) 25:395–7. doi: 10.1097/MBC.0000000000000052 24469392

[50] Michiels JJ Budde U van der Planken M van Vliet HHDM Schroyens W Berneman Z. Acquired Von Willebrand Syndromes: Clinical Features, Aetiology, Pathophysiology, Classification and Management. Best Pract Res Clin Haematol (2001) 14:401–36. doi: 10.1053/beha.2001.0141 11686107

[51] Federici AB Stabile F Castaman G Canciani MT Mannucci PM. Treatment of Acquired Von Willebrand Syndrome in Patients With Monoclonal Gammopathy of Uncertain Significance: Comparison of Three Different Therapeutic Approaches. Blood J Am Soc Hematol (1998) 92:2707–11. 9763553

[52] Wolfe Z Lash B. Acquired Von Willebrand Syndrome in IgM Monoclonal Gammopathy as the Presentation of Lymphoplasmacytic Lymphoma. Case Rep Hematol (2017) 2017:9862620. doi: 10.1155/2017/9862620 28695028 PMC5485300

[53] Rossi D Paoli LD Franceschetti S Capello D Vendramin C Lunghi M. Prevalence and Clinical Characteristics of Immune Thrombocytopenic Purpura in a Cohort of Monoclonal Gammopathy of Uncertain Significance. Br J Haematol (2007) 138:249–52. doi: 10.1111/j.1365-2141.2007.06633.x 17535272

[54] Shimanovsky A Alvarez AJ Murali S Dasanu CA. Autoimmune Manifestations in Patients With Multiple Myeloma and Monoclonal Gammopathy of Undetermined Significance. BBA Clin (2016) 6:12–8. doi: 10.1016/j.bbacli.2016.05.004 PMC4900299 27331023

[55] Merlini G. AL Amyloidosis: From Molecular Mechanisms to Targeted Therapies. Hematol Am Soc Hematol Educ Program (2017) 2017:1–12. doi: 10.1182/asheducation-2017.1.1 PMC6142527 29222231

[56] Sidana S Larson DP Greipp PT He R McPhail ED Dispenzieri A. IgM AL Amyloidosis: Delineating Disease Biology and Outcomes With Clinical, Genomic and Bone Marrow Morphological Features. Leukemia (2019) 34:1373–82. doi: 10.1038/s41375-019-0667-6 PMC8019395 31780812

[57] Grogan M Dispenzieri A Gertz MA. Light-Chain Cardiac Amyloidosis: Strategies to Promote Early Diagnosis and Cardiac Response. Heart (2017) 103:1065–72. doi: 10.1136/heartjnl-2016-310704 PMC5566095 28456755

[58] Sucker C Hetzel GR Grabensee B Stockschlaeder M Scharf RE. Amyloidosis and Bleeding: Pathophysiology, Diagnosis, and Therapy. Am J Kidney Dis (2006) 47:947–55. doi: 10.1053/j.ajkd.2006.03.036 16731289

[59] Cao X-X Meng Q Mao Y-Y Su W Zhen J-F Shen K-N. The Clinical Spectrum of IgM Monoclonal Gammopathy: A Single Center Retrospective Study of 377 Patients. Leuk Res (2016) 46:85–8. doi: 10.1016/j.leukres.2016.05.002 27232065

[60] Mauermann ML. The Peripheral Neuropathies of POEMS Syndrome and Castleman Disease. Hematol Oncol Clin North Am (2018) 32:153–63. doi: 10.1016/j.hoc.2017.09.012 29157616

[61] Dispenzieri A. POEMS Syndrome: 2019 Update on Diagnosis, Risk-Stratification, and Management. Am J Hematol (2019) 94:812–27. doi: 10.1002/ajh.25495 31012139

[62] Swerdlow SH Campo E Harris NL Jaffe ES Pileri SA Stein H. WHO Classification of Tumours of Haematopoietic and Lymphoid Tissues. Lyon: IARC (2017).

[63] Gertz MA. Waldenström Macroglobulinemia Treatment Algorithm 2018. Blood Cancer J (2018) 8:40–0. doi: 10.1038/s41408-018-0076-5 PMC5928091 29712895

[64] Schuster SR Rajkumar SV Dispenzieri A Morice W Aspitia AM Ansell S. IgM Multiple Myeloma: Disease Definition, Prognosis, and Differentiation From Waldenstrom's Macroglobulinemia. Am J Hematol (2010) 85:853–5. doi: 10.1002/ajh.21845 PMC3736855 20842638

[65] Treon SP Xu L Yang G Zhou Y Liu X Cao Y. MYD88 L265P Somatic Mutation in Waldenstrom's Macroglobulinemia. N Engl J Med (2012) 367:826–33. doi: 10.1056/NEJMoa1200710 22931316

[66] Varettoni M Arcaini L Zibellini S Boveri E Rattotti S Riboni R. Prevalence and Clinical Significance of the MYD88 (L265P) Somatic Mutation in Waldenstrom's Macroglobulinemia and Related Lymphoid Neoplasms. Blood (2013) 121:2522–8. doi: 10.1182/blood-2012-09-457101 23355535

[67] Pika T Hegenbart U Flodrova P Maier B Kimmich C Schönland SO. First Report of Ibrutinib in IgM-Related Amyloidosis: Few Responses, Poor Tolerability, and Short Survival. Blood (2018) 131:368–71. doi: 10.1182/blood-2017-09-806463 29180400

[68] Bou Zerdan M Valent J Diacovo MJ Theil K Chaulagain CP. Utility of Bruton's Tyrosine Kinase Inhibitors in Light Chain Amyloidosis Caused by Lymphoplasmacytic Lymphoma (Waldenström's Macroglobulinemia). Adv Hematol (2022) 2022:1182384. doi: 10.1155/2022/1182384 35096069 PMC8791721

[69] Castillo JJ Advani RH Branagan AR Buske C Dimopoulos MA D'Sa S. Consensus Treatment Recommendations From the Tenth International Workshop for Waldenström Macroglobulinaemia. Lancet Haematol (2020) 7:e827–37. doi: 10.1016/S2352-3026(20)30224-6 33091356

[70] Treon SP Meid K Gustine J Yang G Xu L Liu X. Long-Term Follow-Up of Ibrutinib Monotherapy in Symptomatic, Previously Treated Patients With Waldenström Macroglobulinemia. J Clin Oncol (2021) 39:565–75. doi: 10.1200/JCO.20.00555 PMC8078354 32931398

[71] Hughey CT Brewer JW Colosia AD Rosse WF Corley RB. Production of IgM Hexamers by Normal and Autoimmune B Cells: Implications for the Physiologic Role of Hexameric IgM. J Immunol (1998) 161:4091–7. 9780180

[72] Petrušić V Živković I Stojanović M Stojićević I Marinković E Dimitrijević L. Hexameric Immunoglobulin M in Humans: Desired or Unwanted? Med Hypotheses (2011) 77:959–61. doi: 10.1016/j.mehy.2011.08.018 21903335

